# In Vivo Tracking of Tissue Engineered Constructs

**DOI:** 10.3390/mi10070474

**Published:** 2019-07-16

**Authors:** Carmen J. Gil, Martin L. Tomov, Andrea S. Theus, Alexander Cetnar, Morteza Mahmoudi, Vahid Serpooshan

**Affiliations:** 1 Wallace H. Coulter Department of Biomedical Engineering, Emory University School of Medicine and Georgia Institute of Technology, Atlanta, GA 30322, USA; 2 Precision Health Program, Michigan State University, East Lansing, MI 48824, USA; 3 Department of Radiology, Michigan State University, East Lansing, MI 48824, USA; 4 Department of Pediatrics, Emory University School of Medicine, Atlanta, GA 30309, USA; 5 Children’s Healthcare of Atlanta, Atlanta, GA 30322, USA

**Keywords:** in vivo imaging, tissue engineering, 3D bioprinting, additive manufacturing, scaffold tracking, magnetic resonant imaging (MRI), computed tomography (CT), ultrasound, fluorescence spectroscopy, bioluminescence, optical coherence tomography, photoacoustic imaging, magnetic-particle imaging, multimodal imaging

## Abstract

To date, the fields of biomaterials science and tissue engineering have shown great promise in creating bioartificial tissues and organs for use in a variety of regenerative medicine applications. With the emergence of new technologies such as additive biomanufacturing and 3D bioprinting, increasingly complex tissue constructs are being fabricated to fulfill the desired patient-specific requirements. Fundamental to the further advancement of this field is the design and development of imaging modalities that can enable visualization of the bioengineered constructs following implantation, at adequate spatial and temporal resolution and high penetration depths. These in vivo tracking techniques should introduce minimum toxicity, disruption, and destruction to treated tissues, while generating clinically relevant signal-to-noise ratios. This article reviews the imaging techniques that are currently being adopted in both research and clinical studies to track tissue engineering scaffolds in vivo, with special attention to 3D bioprinted tissue constructs.

## 1. Introduction

A significant portion of recent advancements in the field of tissue engineering (TE) has focused on design, developing, and characterization of new biomaterials that can be used as tissue mimics to model a variety of diseases in vitro, or as implants to repair or regenerate damaged tissues in vivo [1,2,3]. Further, the advent of new automated additive manufacturing techniques, such as 3D printing and bioprinting, together with computer-aided design (CAD) modeling, have allowed for higher throughput biofabrication of 3D scaffolding systems with increasing structural and functional complexities to be used in patient-specific TE and precision medicine applications [4,5,6,7,8]. Thus, it is vital to design and utilize effective imaging and tracking methods to closely monitor the scaffolds following implantation in the patient’s body [9,10]. These techniques should enable noninvasive, real-time examination of properties including the graft stability and position, biomaterial-tissue interactions (e.g., biocompatibility, degradation, and integration with host tissue), blood perfusion (angiogenesis), and function (e.g., contractile function of a cardiac patch). To achieve this goal, imaging techniques with minimal invasiveness as well high penetration depth and high resolution are required to provide a clear contrast between the embedded biological materials and the surrounding tissue structure, thus generating a complete picture covering morphological, physiological, and molecular processes [11].

**Figure 1:**
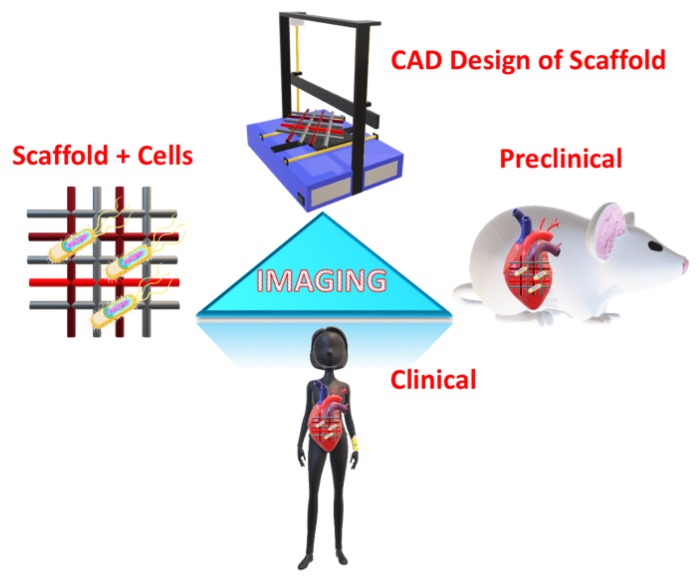
Schematic illustration for the role of imaging in tissue engineering (TE) applications at different levels: scaffold design using computer-aided design (CAD), cellular scaffolds in in vitro applications, preclinical application through implantation in animal models, and clinical application in humans.

New advancements in medical imaging have been made to address different challenges in the TE field, ranging from the design to production processes of tissues and organs, as well as clinical implantation and implementation (Figure 1) [10,12]. These imaging systems often follow a common process of exciting the targeted samples with an energy source like electromagnetic radiation, light, or sound, or a combination of those sources, to generate a response in the form of emitted, transmitted or reflected signal which can then be captured through different detector designs for analysis [10]. Furthermore, specific techniques also require contrast agents or sample labeling methods to enhance the signal-to-noise ratio [13]. As a result, different methods would have distinct advantages and disadvantages with respect to the penetration depth, image temporal and spatial resolution, as well as the effect of the exciting source and contrast/labeling agent on the biological target(s) (Table 1). Thus, it is important that the techniques are selected carefully and tailored to fulfill the specific application requirements.

In this review, we detail the different medical imaging techniques used in TE applications. These methods include: computed tomography (CT), magnetic resonance imaging (MRI), magnetic particle imaging (MPI), ultrasound, photoacoustic imaging, different optical methods (fluorescence spectroscopy, bioluminescence, and optical coherence tomography), and multimodal imaging (Table 1). These methods have been widely applied in the different areas of TE for investigating the morphological structures of the scaffold structures as well as studying the viability of the different cellular constructs [11]. The Main focus will be on delineating how the general principle of operation and recent advancements in the imaging methods can aid the researchers in the field to select the most effective imaging methods for their in vivo studies.

## 2. Scaffold Tracking Techniques

### 2.1. Magnetic Resonance Imaging (MRI)

**Figure 2:**
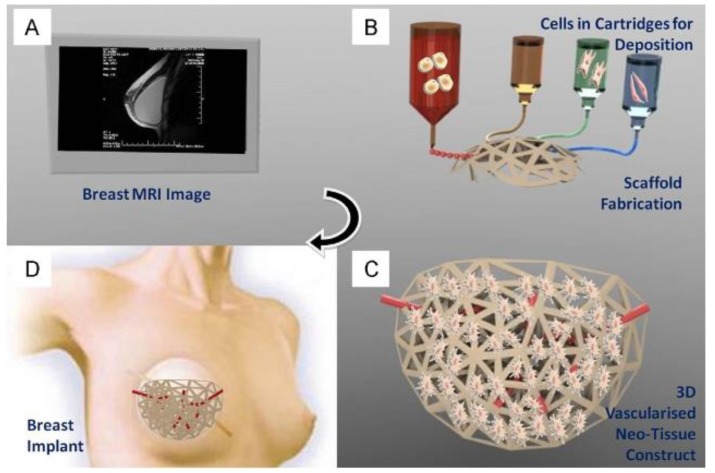
Application of patient’s MRI data to generate a bioprinted scaffold for organ regeneration, disease treatment, or drug delivery. MR images (**A**) of the target organ/tissue will be acquired and processed to create a 3D STL file. The model will be 3D bioprinted using various inks and scaffolds (**B**), cultured in vitro to establish the new tissue structure and vasculature (**C**), followed by implantation in vivo to repair/regenerate target tissue/organ (**D**). Reproduced with permission from Ref. [19].

Magnetic resonance imaging (MRI) is a commonly used imaging method that has broad applications in the clinical and basic research fields. Briefly, MRI is a non-invasive imaging technique that allows for pertinent information to be gathered over the entire patient body in a highly detailed manner [14]. Importantly, MRI is usually not associated with harmful radiations, allowing for repetitive scans and longitudinal studies to be performed with minimal harmful effects, which further makes this imaging method an attractive way to diagnose clinical pathologies [15]. It uses magnetic fields and radio waves often combined with contrast agents to generate highly detailed images of tissues within the body and is widely used for applications in the clinics. Recently, there has been an increasing interest for the use of MRI in various TE applications, because of the capability of the technique in producing high-resolution 3D structural scans with minimal damage to the tissue mimic [16,17,18]. Such 3D reconstructions can be used, via 3D bioprinting methods, to create patient and disease-specific tissue constructs for regenerative therapies (Figure 2).

Recent advances in contrast agent design and development have made it possible to detect and track cell populations within the complex tissue-engineered constructs both in vitro and post implantation in vivo. These in vivo MRI cell-tracking processes can be performed using a variety of contrast agents, such as gadolinium, fluorine, or manganese, as well as superparamagnetic nanoparticles [20,21]. These materials are the preferred MRI contrast agents for TE applications as they are usually less cytotoxic and offer more reliable cellular uptake for imaging. In addition, MRI used in conjunction with different functionalized nano-contrast agents and other imaging techniques has also been used to study different drug release kinetics in the field of tissue engineering [22,23,24]. To date, MRI imaging is increasingly used in conjunction with the next-generation additive manufacturing technologies, such as bioprinting, to track cells in TE scaffolds. MRI has been successfully used as both a diagnostic and tracking tool, which readily allows for translation of in vitro imaging processes in the basic research stage to the clinical settings [19,25].

**Figure 3:**
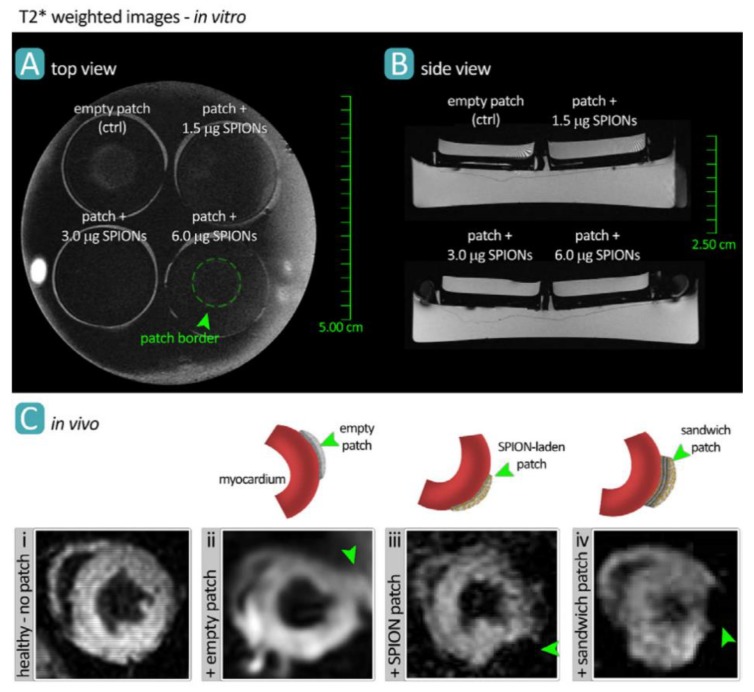
MR imaging of bioengineered collagen constructs used as cardiac patch to repair ischemic heart tissue. Patches were loaded with 1.5, 3.0, and 6.0 μg/mL of iron oxide nanoparticles and imaged via MRI both in vitro (**A**,**B**) and in vivo (**C**), in a mouse model. Manganese-enhanced MRI visualized the patch grafted onto healthy myocardial tissue in different groups including no treatment (control) (**i**), empty patch (**ii**), nanoparticle-loaded patch (**iii**), and loaded-empty-loaded sandwich patch (**iv**). Reproduced with permission from Ref. [17].

For instance, superparamagnetic nano-sized iron oxide particles, coated with polyethylene glycol (PEG), have been used to label both rat and human T-cells in vivo, with over 90% efficiency and without any measurable effects on T-cell properties [26]. Iron oxide nanoparticles were used in another study as an MRI contrast agent, to label and track collagen-based cardiac patches following implantation onto the epicardial surface of the mouse heart (Figure 3) [17,18,27,28]. T2*-weighted MR images demonstrated the robust capability of this technique to noninvasively visualize the engineered patch device.

Stem cell-derived cellular cultures used in cartilage [29], adipose [30] and heart TE [31] have also successfully used MRI in the development, characterization, and clinical translation of the scaffolding constructs. Furthermore, MRI imaging with superparamagnetic iron oxide nanoparticles as exogenous labeling agents has been used to study different stem cell dynamics for both preclinical and clinical applications [32]. As a result, with the rapid growth of various stem cell therapies, clinical MRI will be more extensively used as a robust, noninvasive bedside tool for guided administration, delivery, and tracking of transplanted cells [33]. There is also a small but significant body of work that applied MRI imaging to nanoparticle vaccine efficacy, focusing on immune system priming and cellular activation in cancer vaccine development [34].

### 2.2. Magnetic Particle Imaging (MPI)

**Figure 4:**
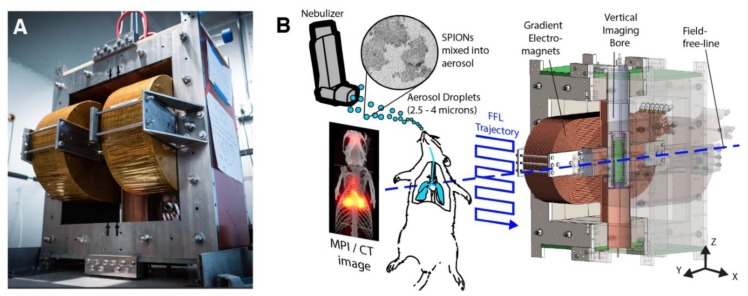
Hardware setup used in magnetic particle imaging (MPI) scans using SPIONs. (**A**) The Berkeley field-free-line MPI preclinical scanner. (**B**) To form a projection image, the magnetic field (FFL) rasters across a trajectory as shown, imaging the in vivo distribution of SPIONs in a rat. Multiple such projections can reconstruct a 3D MPI image similar to CT. Reproduced with permission from Ref. [48]**.**

Apart from MRI, superparamagnetic iron oxide nanoparticles (SPIONs) have also been used in an emergent imaging technique such as magnetic particle imaging (MPI) through their strong magnetization (Figure 4) [35,36,37]. MPI, in conjunction with MRI as a paired technique, is gaining traction in clinical diagnostics due to its significant benefits over other more established techniques [36,37,38]. Specifically, MPI is a relatively fast imaging method, generates zero tissue background signal, and there is no attenuation of the signal correlated to organ depth, allowing for unimpeded and quantitative high-resolution imaging at any depth and location [38,39,40]. Recent work has advanced the technique in its potential for robust and sensitive cardiovascular imaging of healthy and diseased conditions, such as stenosis and myocardial infarcts [39,41,42] and cell tracking [43,44,45,46,47], which is of great interest in the field of TE. In particular, MPI can be useful in imaging bioprinted organ constructs, where 3D spatial arrangement and resolution are fundamental limitations. Further progress in MPI, as a diagnostic and basic research tool, requires advancements in imaging physics, nanoparticle synthesis, and characterization, as well as ongoing proof-of-principle imaging of small animals, TE constructs, and more broadly in human patients [48].

MPI works are based on the direct imaging of the concentration and location of the SPION tracers, using varying magnetic fields, which have high sensitivity and are capable of significant background to signal (contrast) resolution. The field has already advanced several particles (e.g., Ferumoxytol) through the FDA for chronic kidney disease induced anemia treatment [49]. Additionally, SPIONs have been shown to successfully work in patient imaging (Resovist) [50,51], and have been used to localize sentinel lymph nodes for breast cancer detection (Sienna) [52]. They have been also used for evaluation of hyperthermia-induced solid tumor removal (NanoTherm) [53]. Importantly, MPI scans are relatively safe and radiation-free, which combined with the high contrast and sensitivity imaging capabilities, offers critical advantages in cardiovascular imaging and cell tracking. MPI has been used both as a diagnostic tool and as an imaging platform in bioprinting-based TE, to build tissue/organ mimics with high fidelity to their living analogs. It is capable of detecting low numbers of cells reliably [54], which opens up the technology to be used in cell-based clinical applications, such as cancer therapies and tracking stem cell-derived TE constructs [43,46,52]. Unlike traditional radiotracing contrasts, SPIONs have a half-life that is essentially unlimited, which allows researchers to track cellular localization over long time intervals (up to several months in animal models) [55].

### 2.3. Angiography

**Figure 5:**
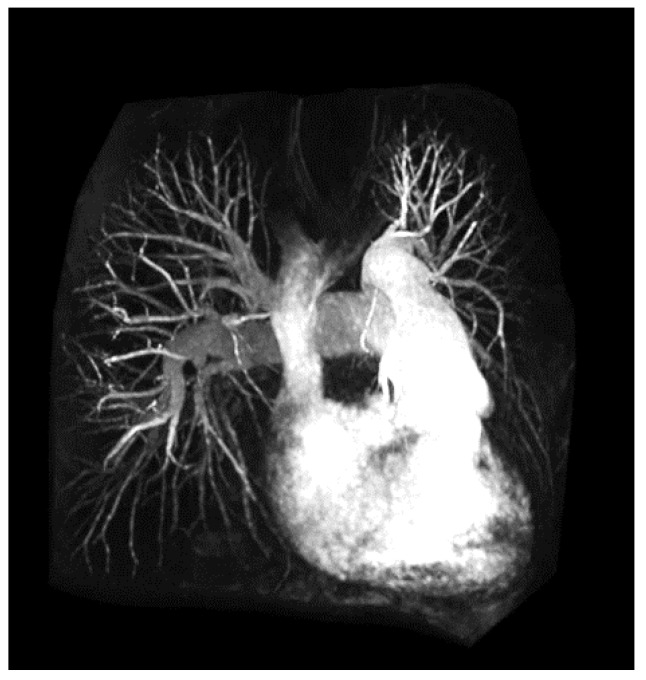
Example angiography (MR) outlining via contrasting the heart chambers and attendant vasculature. Reproduced with permission from Ref. [64].

An angiogram or arteriogram is a diagnostic procedure that uses specific dyes to outline the arteries in a patient (Figure 5). Arteries are invisible to the clinical imaging tools under normal conditions, and thus, their visualization requires utilizing some type of contrast agents. There are three main forms of angiograms, each relating to the imaging platform that is used to generate the clinical images.

Digital subtraction angiography (DSA) is the more common method of getting arterial images in the clinic. The artery to be imaged is numbed with a local anesthetic and then a contrast agent is injected, which outlines the vascular network downstream. Following contrast introduction, X-ray is used to acquire the vasculature images [56,57,58,59]. DSA normally takes around 20 min to perform. Computer tomography (CT) angiography is another method to acquire high-resolution 3D images of a patient’s vasculature. Similar to DSA, CT angiography also requires a contrast agent introduction, but unlike DSA, the injection site is the vein in the arm, usually a drip, which allows for the entire arterial network to be imaged, if required. Image acquisition is very fast as it only takes a few seconds to generate them. The third common angiography method uses MR to generate the needed 3D vasculature images [58,60,61,62,63,64]. Gadolinium is the most common contrast agent that is used with MR angiography (Figure 3). Post introduction, any artery in the body can be imaged. As with CT angiography, MR approach is a fast procedure, usually performed on the same day.

Since angiography procedures involve the injection of a contrast agent to generate images, there may be some risks, such as allergies to the contrast, bleeding at the puncture site, or false aneurism [65]. A rarer but serious complication can happen if there is already some kidney damage present, where contrast injection can further deteriorate kidney function. Each of these complications can be successfully mitigated via appropriate pre-procedure preparations, or with simple surgical post-procedure manipulation in the case of the false aneurysm.

Angiography is almost exclusively a clinical imaging technique, so its application to in vitro TE has been limited so far, done predominantly in excised tissue slices from mice and pig. Nonetheless, as the additive manufacturing, specifically bioprinting technologies, enter the tissue bioengineering field, complex in vitro tissue models that incorporate vascularization will require advanced visualization and tracking methods for both modeling applications in the lab and for translational applications, such as cardiac patch implants or vessel grafts post-stenosis. Having a complete picture of all sources of flow into and out of bioengineered tissue mimics would be critical to recapitulating their functionality.

### 2.4. Computed Tomography (CT)

**Figure 6:**
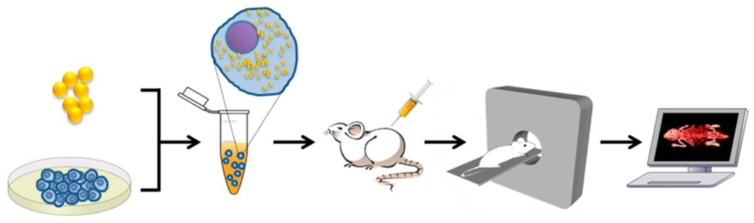
Cellular tracking using gold nanoparticles as a contrast agent and imaged with CT. Reproduced with permission from Ref. [77].

CT has been widely used as a biomedical imaging technique over the last decades due to its high spatial and temporal resolution. CT imaging generates a 3D reconstruction of the targeted sample by collecting the transmitted X-ray at different angles using a multi-array detector (Figure 6) [12]. Since the CT contrast is sensitive to the materials that attenuate the X-ray transmission, this technique has been widely used to image tissue structures which have high mineral concentrations, like bone and the surrounding tissue. Consequently, CT has been extensively used in different bone TE applications [66,67,68,69]. The development of more sensitive techniques like micro-CT has allowed for the study of the morphology and 3D structure of different scaffold geometries in the sub-micron scale as well as the tracking of different cells incorporated into the scaffold structures [70,71,72,73,74,75,76]. These unique advantages have also allowed CT to be used in conjunction with new additive manufacturing techniques, such as 3D (bio)printing, for different implant-manufacturing purposes [66].

A drawback of the CT technique is that it is less sensitive to visualize the contrast between different soft tissue structures. However, the sensitivity can be improved by utilizing different contrasting agents. Currently, different biomaterials including gold [78,79,80,81,82,83], heavy elements [84], cationic agents [85], polymers [74], and nanoparticles [84,86] are being used as contrast agents in different CT imaging applications ranging from animal models to clinical studies. For instance, different animal model studies have employed CT with radio-transparent contrast agents like polymer [87] and alkaline-based agents [88] as in vivo imaging techniques to quantify different soft tissue structures like hepatic vascular and parenchymal regeneration as well as vascular network at a capillary level. CT with contrast angiography has also been used to study the stability of human cell-derived engineered heart valve after implantation in sheep [89,90]. Clinically, CT with an iodine-based agent has been used to study myocardial fibrosis in patients with hypertrophic cardiomyopathy [90]. These materials can be implanted in the soft tissue scaffold, thus allowing for new in vivo tracking applications. Furthermore, these nanobiomaterials can also be used as therapeutics by including functionalized medicine through surface modification [77,91]. In addition to the development of new biocompatible contrasting materials, more sensitive detectors such as photon-counting detector technology have also been developed to enhance the visualization of different soft tissues within the CT techniques [92]. As a result, contrast agents can be used both as diagnosis and therapeutics. Thus, CT imaging technologies can be used to monitor the efficacy of drug implants in vivo due to its high spatial resolution and penetration in comparison with other imaging techniques.

More recently, CT imaging has been utilized as a nondestructive tool for longitudinal and volumetric measurement of scaffold degradation both in vitro and in vivo [81]. For this purpose, gold nanoparticles, used as contract agents, were covalently conjugated to collagen polymer during scaffold fabrication, resulting in the generation of CT-visible collagen constructs. The X-ray attenuation of the conjugated scaffolds was used to measure hydrogel degradation over the time in culture.

### 2.5. Ultrasound

The rapid development of implantable TE platforms has created a major necessity for non-invasive, non-ionizing, and non-destructive techniques for the in vivo tracking and imaging of implantable tissues. Ultrasound imaging technologies and their associated multi-modality approaches (e.g., ultrasound-photoacoustic imaging [93]) have been investigated due to their specific advantages for TE applications (Figure 6). Importantly, ultrasound techniques can enable in-situ quantitative measurements of various properties of engineered tissues, including extracellular matrix (ECM) formation, degradation, mechanical strength, cell infiltration, vascularization, and blood perfusion and oxygenation [93,94]. This is while most in vitro or ex-vivo scaffold characterization modalities, such as electron or optical microscopy, and X-ray tomography have limitations for in vivo tracking of scaffolds, due to their invasiveness, limited penetration depth (few hundred micrometers), or poor contrast. Thus, ultrasound could be an ideal tool for diverse preclinical and clinical applications.

**Figure 7:**
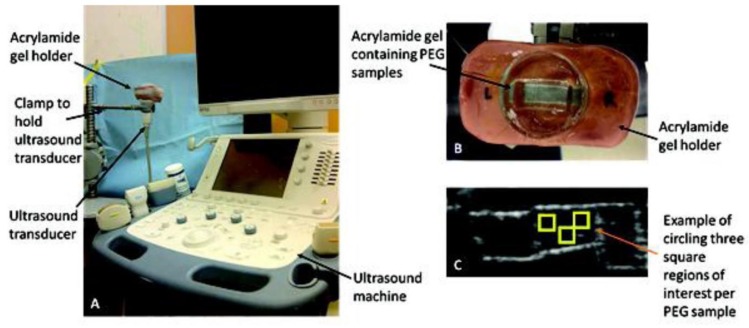
(**A**,**B**) Experimental setup of ultrasound used to image different areas of a scaffold made of PEG hydrogel. (**C**) Example of output image from the system. Reproduced with permission from Ref. [96]**.**

Ultrasound utilizes sound waves at frequencies over 20 kHz (Figure 7). In a clinical setting, frequencies ranging from 1–15 MHz are used to generate images of features in biological tissues [95]. For instance, PEG hydrogels have been characterized using B-mode ultrasound to visualize varying amounts of ECM proteins and cell composition in real time over 18 days [96]. This technique utilizes a 12 MHz imaging frequency which limits the penetration, making it difficult for probing scaffolds in vivo. However, this method is suitable for validation experiments in preclinical applications. This validation method was used in a similar study, where collagen deposition was calculated in scaffolds with myofibroblasts to quantify protein concentration [94,97,98,99].

Along with quantifying biological components concentration, mechanical properties of TE constructs can be evaluated using ultrasonic modalities coupled with computational methods. Previous studies have shown that utilizing ultrasound elastography can yield measurements of elasticity and stiffness of soft tissues to determine pathological conditions such as inflammation and tumors. For instance, Walker et al., reported a strong correlation between the compressive moduli calculated from ultrasound and mechanical testing of TE cartilage [100]. Ultrasonic modalities offer robust information due to their unique interactions with biological tissues. As effective signal processing and computational methods improve, more methods are increasingly being applied to generate information-rich data sets of TE tissues from ultrasound acquisitions.

While ultrasound (sonography) methods may offer much lower resolution than MRI and CT for imaging of bioprinted constructs, they can monitor the condition of bioprinted tissues in vivo in real time readily, at much lower costs [101]. Ultrasound has recently found other novel applications in 3D bioprinting technologies, helping to tackle some of the main challenges related to this additive biomanufacturing techniques. Acoustic radiation in an ultrasound standing wave field (USWF) forces the cells and accumulates them at the pressure nodes, at low pressure areas [101]. This technique (so called ultrasound-assisted biofabrication) results in the formation of cell spheroids within minutes at relatively narrow/homogeneous size distributions. For instance, USWF was utilized to generate endothelial cell spheroids which showed enhanced neovessel formation [102]. Further, low-intensity ultrasound is reported to enhance stem cells proliferation and differentiation [103].

### 2.6. Bioluminescence

Bioluminescence is a natural light-emitting process, produced by various organisms, which can provide a measure of localization and viability of cells and tissues [104,105,106]. Derived from bioluminescent species, the light-emitting oxidation reaction of luciferase (enzyme) catalysis of luciferin (substrate) can be introduced to cells. Once a substrate is introduced, transfection of luciferase-coding genes into cells primes them for light production. The light intensity can then be detected and quantitatively evaluated—known as bioluminescence imaging (BLI) [104,107]. Resolutions of BLI can accurately trace down to the molecular or cellular scale across entire organisms during in vivo tracking [104,105]. Unlike potentially harmful contrast agents, this illumination process is non-invasive, biologically compatible, and can be longitudinally monitored across cell lineages. It also avoids the high cost, low throughput, and low sensitivity of standard instrumentation, such as MRI or CT. However, current challenges include deep tissue visualization, spatial resolution, homogeneous substrate distribution, and accurate interpretation of detected signals [104,108].

Cell tracking via BLI has been used for stem cells, immune cells, and bacteria for varied tissue types [104]. For instance, using human mesoangioblasts, seeded onto decellularized esophageal scaffolds, Crowley et al. quantified cell viability, proliferation, and migration after implantation in murine models over the course of 7 days [109]. Iwano et al. visualized tumorigenic cells in deep mouse lung vasculatures and also demonstrated successful tracking of hippocampal neuronal activity, while previous luciferins were too large to penetrate [110]. Notably, these longitudinal studies have been carried out for up to 16 months in marmosets [111]. To study the immune response, Conradi et al. monitored a fibrin scaffold seeded with neonatal rat heart cells when implanted in allogeneic, syngeneic, and immunodeficient rat recipients [108]. Allogenic grafts only survived for 14 ± 1 days, while the syngeneic and immunodeficient recipients lasted over 100 days, indicating the importance of autologous cell sourcing. Ex-vivo validation and improvement of BLI is continually being performed to advance in vivo techniques as well, such as stem cell seeding on intervertebral discs in culture [112]. Therefore, BLI is known as a longitudinal, non-invasive method of tracking implanted tissues and their progeny in vivo.

To improve the tissue penetration in vivo, synthetic enzyme and substrate analogues have emerged that emit longer, near-infrared (NIR) wavelengths (λ = 650–900 nm) that are unhindered by hemoglobin and melanin absorption ranges (λ ≤ 600 nm) [110]. Used luciferases originate from fireflies, sea pansies, and photobacteria, but have relatively similar maximum emission spectrums (λ ≤ 600 nm) [104]. Luciferin analogues have also been explored. For instance, AkaLumine-HCl has achieved NIR wavelengths (λ = 677 nm) and demonstrated improved spatial sensitivity in deep lung metastases down to the single cell level, as compared to luciferin-D or CycLuc1 [110]. A mutagenic derived luciferase, specific to AkaLumine-HCl, called Akaluc, was also engineered to boost catalysis efficiency by sevenfold. AkaLumine-HCl is permeable through the blood-brain barrier and evenly distributes at low concentrations [111]. Overall, as techniques evolve, the BLI utility in localizing tissue-engineered constructs longitudinally will continue to distinguish this method from other in vivo imaging modalities.

**Figure 8:**
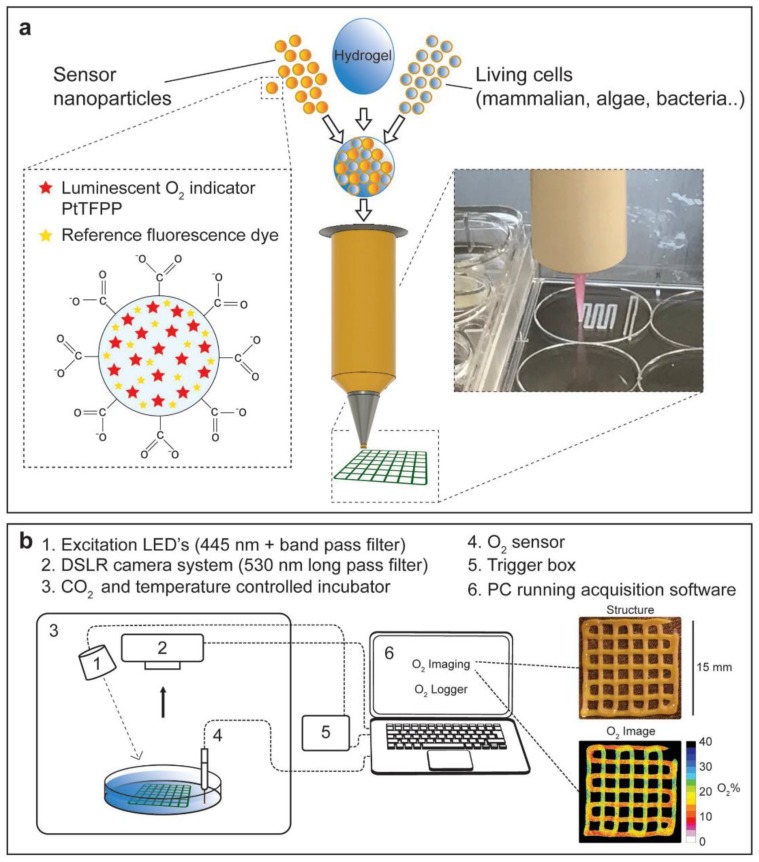
A novel 3D bioprinting approach with hydrogel bioinks functionalized with luminescent nanoparticles. (**a**) Cells and/or nanoparticles, containing the O_2_-sensitive luminescent indicator PtTFPP compound and an inert fluorescent coumarin dye, were incorporated into an alginate-based bioink for bioprinting. (**b**) Experimental setup used to image O_2_ distribution in bioprinted hydrogel constructs.

In a recent approach, functionalized bioinks were developed by incorporating luminescent optical sensor nanoparticles into the hydrogel ink solution [113]. Excitation of these nanoparticles with blue light results in the emission of red luminescent light by the particles which is in proportion to the local oxygen concentration. Higher oxygen contents will generate less red luminescence. Therefore, this innovative, noninvasive approach enables imaging and analysis of the heatmap of red luminescence and oxygen concentration within bioprinted tissue constructs (Figure 8) [113]. In another study, the proliferation of bioprinted mesenchymal stromal cells, associated with collagen and nano-hydroxyapatite, was assessed by quantification of the luciferase signal of luciferase positive cells in a mouse model for up to 42 days [114].

### 2.7. Fluorescence Spectroscopy

Fluorescence spectroscopy utilizes the ability of the targeted molecules to emit light at a different wavelength than the optical excited source (Figure 8) [115]. The information from the emitted photon can then be constructed to produce 2D images with high temporal and spatial resolution [116]. In biology, fluorescence can happen with most biological molecules with the appropriate excitation. However, for specific applications, like cell-based therapy and TE, the emitted signal from the fluorophore molecules can be enhanced through direct or indirect labeling. In the case of indirect labeling, the targeted cells can be engineered, like gene transfection, to express fluorescence proteins like GFP [117]. In indirect labeling, the targeted molecules are attached to certain functional fluorescent molecules which can be activated through an optical excited source [118]. In both cases, the excited wavelength needs to be considered carefully since it can affect the specific photophysical properties of the fluorophores, such as photostability, quantum yield, Stokes shift, and fluorescence lifetime [118].

**Figure 9:**
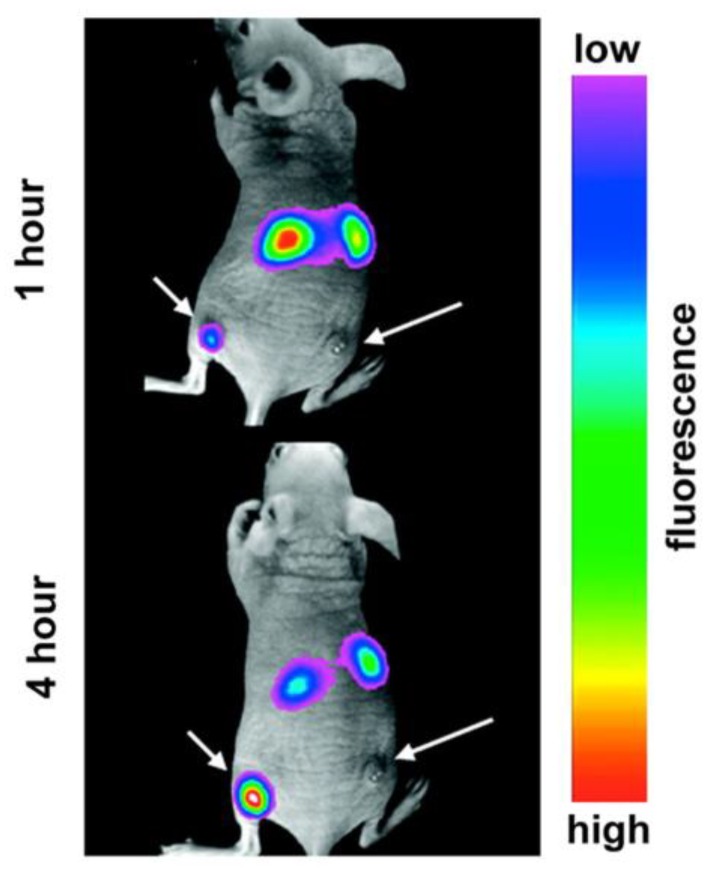
Fluorescence contrast of tumor growth when the mouse is injected with upconversion nanoparticles. Reproduced with permission from Ref. [115].

Nanomaterials have emerged as an effective candidate for direct labeling in fluorescent spectroscopy. Different materials such as quantum dots (QDs) [119], polymers [115,120,121], organic dyes [122], upconversion nanoparticles (UCNPs) [123], and gold nanoparticles (AuNPs) [115] have been considered depending on the specific applications. For cellular tracking, the functional nanomaterials can be attached ex vivo to the targeted protein on the cell surface or they can be infused inside through the process of diffusion or active transport [118]. When excited optically, these nanomaterials can be used to distinguish different performance and functionality of the matrix-embedded cells. In addition to cellular studies, nanomaterials, such as QDs [116] and UCNPs [123], have also been used to label hydrogel scaffold structures to study their degradation. These materials require different excitation wavelengths such as ultraviolet (UV) or visible light for QDs and dyes [115], while UCNPs are more sensitive to NIR wavelengths [123]. However, UV and visible wavelengths have different shortcomings, such as limited penetration depth and potential disintegration of the biological molecules and scaffolds [118]. In addition, even though QDs have excellent optical properties, their suboptimal biocompatibility and biodegradability represent major challenges that hinder their applications in TE [115]. NIR fluorophores can resolve those disadvantages as well as minimize the autofluorescence from cells and tissues, which allows them to be used in a wide range of in vivo tracking of hydrogel degradation (Figure 9) [115].

In addition to fluorescent properties, certain metallic nanoparticles like gold nanoparticles can have multi-functional properties, contributing to tissue mechanical properties, electrical conductivity and (cell) differentiation, and photothermal effect, when they are integrated into the biopolymer scaffold. Such particles, therefore, are good candidates for multifunctional applications such as cancer detection assays and optically-controlled on–off microfluidic devices [124].

### 2.8. Optical Coherence Tomography

**Figure 10:**
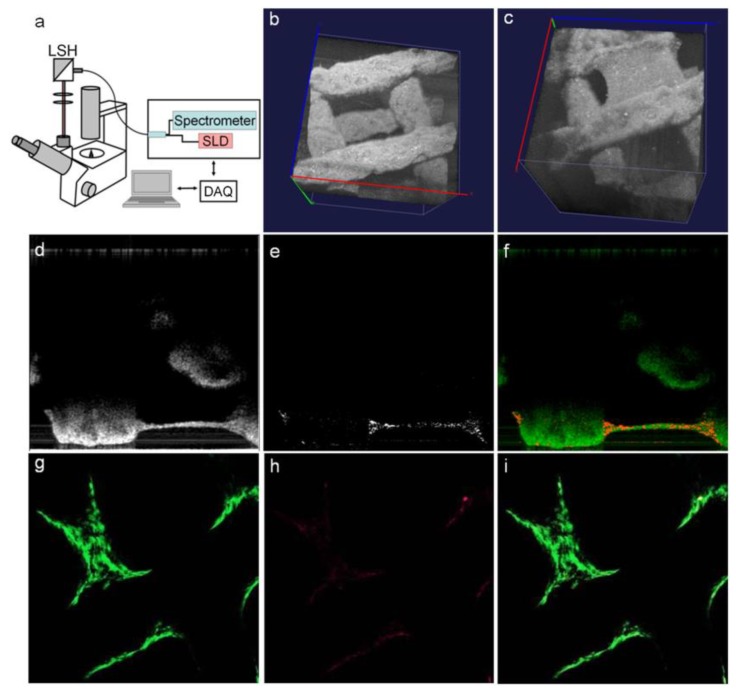
(**a**–**i**) OCT design and the tracking of cells in a 3D bioprinted scaffold seeded with cells. Reproduced with permission from Ref. [127].

In addition to fluorescence and bioluminescence, which only provide 2D image information, recent advances in optical imaging technologies have allowed for the 3D visualization of tissue structure through the measurement of the interference and coherence between signals reflected from the object and reference signals, known as optical coherence tomography (OCT) [11]. Due to this unique property, OCT can provide anatomical information of the object with sub-millimeter penetration depth [10]. OCT can be used with a variety of light sources, ranging from NIR to visible light [125,126]. In the field of TE, OCT has been used to investigate the geometrical parameters of 3D scaffold architecture, including porosity, surface area, pore sizes, and pore interconnectivity [127,128], as well as remodeling and degradation of polymer structures for specific applications such as vascular grafts [129]. OCT can also be used to asses cell viability, proliferation, distribution, morphology, and function within a cell-laden hydrogel and scaffold (Figure 9) [130]. Advancement in phase-based OCT has also been able to provide contrast between cells and the surrounding hydrogels, thus allowing to achieve a greater understanding of the cell–ECM interactions [128]. Furthermore, a combination of OCT with Doppler velocimetry has been used to characterize flow in engineered tissues, such as artificial blood vessels, by increasing the obtained contrast compared with conventional OCT [11]. Thereupon, OCT has been widely used in conjunction with automated 3D fabrication techniques, such as 3D bioprinting [131], as a high-resolution, noninvasive, label-free method, enabling cellular imaging at different levels for TE applications (Figure 10).

### 2.9. Photoacoustic Imaging (PAI)

**Figure 11:**
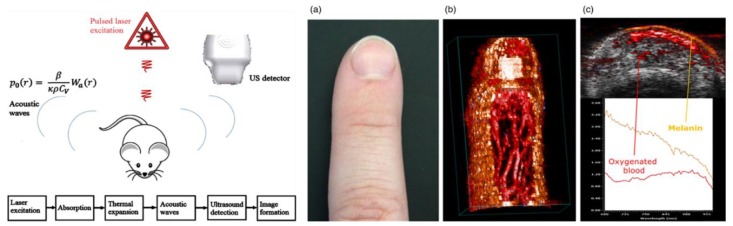
Overview of the physics and processing involved in photoacoustic imaging (PAI) (**left**), and a sample of 2D and 3D vasculatures acquired via PAI from hemoglobin and melanin emissions without contrast agents (**right**, **a**–**c**). Reproduced with permission from Ref. [132].

Photoacoustic imaging (PAI) leverages the photoacoustic effect produced by pulsed non-ionizing lasers in tissue to reconstruct an image. For this method, the pulsed laser energy causes heat-induced, elastic tissue expansion, of which emits ultrasonic waves in the MHz range. Via ultrasound transducers, these waves are detected and electronically processed to output a final picture (Figure 11) [132]. PAI diverges based on the acquisition method into photoacoustic microscopy (PAM—focused scanning) and photoacoustic tomography (PACT—inverse reconstruction) [133]. Overall biomedical applications of this technique vary from visualizing macroscopic structures (e.g., small animals to tissues) to microscopic structures (e.g., cells to organelles), with associated contrast agents [133,134]. The advantages of PAI include its non-invasiveness, non-destructiveness, macro to microscale versatility, and compatibility with established imaging modalities. Conversely, PAI challenges include merging optical and acoustic signals, limited scanning speed for wide fields of motion and optimized mathematical models across measurement scales [132,133].

Applications of PAI in TE and 3D (bio)printing are beginning to expand. For instance, Cai et al., compared the resolution of microcomputed tomography to PAM technique in scaffolds of poly(lactic-co-glycolic acid) incorporated with single-walled carbon nanotubes. They demonstrated commensurate porosity measurements under physiological conditions [134]. In another study, acoustic and physiomechanical properties of 3D bioprinted poly-(ethylene glycol)-diacrylate scaffolds were quantified using an ultrasound pulse echo technique [135]. Hu and Wang have shown micrometer-level resolutions of microvasculatures capable of both capturing geometric and hemodynamic information, such as blood oxygenation [136]. This can be extremely useful in monitoring the oxygenation of implanted TE constructs. Much of PAI research is currently focused on optimizing contrast agents for high fidelity imaging for eventual in vivo applications [137,138,139].

Contrast agents are not always needed due to the PA emissions of already present hemoglobin and melanin. However, to penetrate beyond 1 mm depth, contrast agents that absorb NIR waves are optimal [140]. Metallic (e.g., gold and copper selenide-gold [141]), organic (e.g., carbon tubes and graphene oxide [138]), and semiconductor (e.g., semiconductor polymers and quantum dots [137]) nanoparticles of varying orientations have been employed. Of the three, organic particles exhibit size-independent properties and improved biocompatibility and biodistribution, especially with surface neutralization via encapsulation. This enables multiplexed imaging with customized organic nanoparticles [140]. Overall, PAI development holds much promise in monitoring the TE systems.

### 2.10. Multimodal Imaging

**Figure 12:**
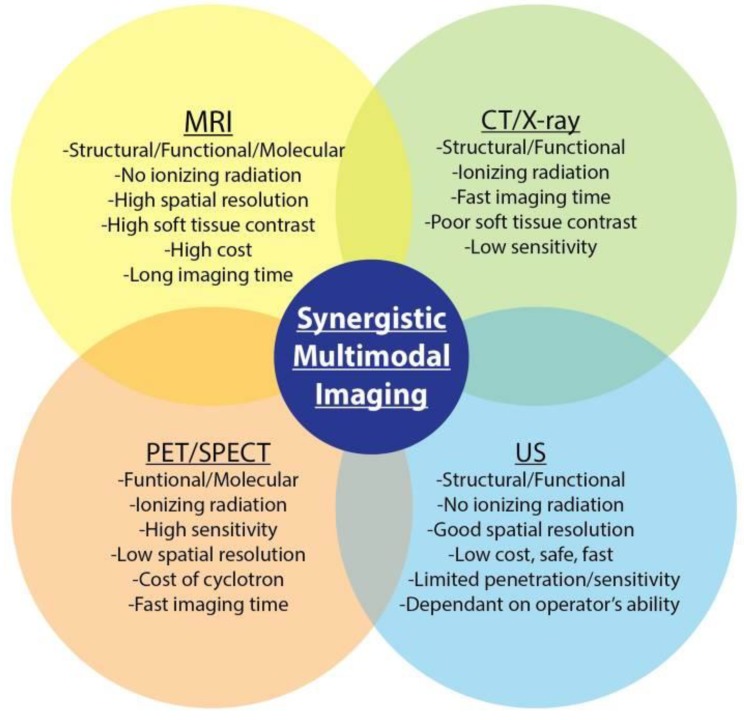
Overview of multimodal imaging applications and their advantages and disadvantages. Reproduced with permission from Ref. [143].

Each imaging modality, described above, is associated with certain drawbacks and hence, a single imaging modality may not be utilized to acquire all desired information from the tissue/construct of interest. Multimodal imaging can be used to overcome these limitations by utilizing a combination of imaging modalities to provide better resolution in terms of spatial information, along with functional and molecular information [142]. Current platforms of multimodal imaging strategies explore combinations of CT/positron emission tomography (PET), CT/MRI, MRI/PET, and ultrasound/PA to characterize and monitor TE constructs (Figure 12) [143].

Contrast agents are often used in multimodal imaging, providing reliable detectability. Radioactive isotopes such as 99mTc (t1/2 = 6 h) and 18F (t1/2 = 110 min) are commonly used in single photon emission CT (SPECT) and PET imaging, since both modalities rely on the detection of ƴ-photons emitted from radioactive isotopes [144,145]. SPECT/CT was utilized to non-invasively monitor bone morphogenetic protein-2 (BMP-2) content and bone formation in composite TE constructs for bone regeneration [146,147]. Kempen et al., created a drug delivery model composed of poly(lactic-co-glycolic acid) embedded into a gelatin hydrogel scaffold over 56 days. A reliable sustained release profile of the 125I-radiolabeled BMP-2 was recorded with SPECT over the full implantation period while in vivo micro-CT detected initial bone formation [146]. However, for multimodal applications, a common contrast agent that each modality can detect is the most favorable. Some nanomaterials such as liposomes, carbon nanotubes, gold nanoparticles, and iron oxide nanoparticles are efficient candidates due to their inherent biocompatibility [17,144,148,149].

Multimodal imaging methods are being increasingly used to track 3D bioprinted tissue constructs. For instance, ultrasound-guided PA imaging technique has shown to have great potential in visualizing the structure, distribution, and retention of microvascular endothelial cells within 3D tissue constructs [150]. Overall, multimodal imaging is dependent on suitable contrast agents that allow for capturing the synergetic properties of each imaging system.

## 3. Conclusions

Imaging techniques have proven to be indispensable to the advancements in the field of TE and regenerative medicine. In vivo imaging and tracking methods provide vital information about different aspects of engineered tissue constructs post implantation. These features include the 3D geometrical microstructure; the interaction between biological molecules and the scaffold; and the cellular behavior, interaction, and viability within the constructs. Acquiring this information is important to provide feedback to improve the design and fabrication of scaffold systems for different clinical applications, as well as to enhance the understanding of certain cellular processes for cell-based therapies. The individual techniques outlined in this study offer different advantages for the in vivo monitoring of molecules and cells. Different methods have distinct capabilities in tracking different properties of 3D scaffolds. These techniques also suffer from distinct disadvantages which can limit their application in clinical trials. Thus, it is important for the researchers to choose the appropriate imaging modality for specific in vivo studies. The emergence of multimodal imaging has provided an alternative to overcome the shortcomings of the individual imaging techniques, thus enabling a more comprehensive visualization at different levels. Furthermore, progresses in developing various contrast agents for different imaging modalities have enhanced the imaging resolution as well as the ability to combine multifunctional contrast agents as both diagnostics and therapeutics. Finally, advanced imaging techniques can also be combined with new fabrication techniques, such as 3D bioprinting, thus allowing for patient-specific therapeutic applications.

## Figures and Tables

**Table 1:** List of non-invasive imaging methods, their resolution and depth, costs, external material usage, information type as well as applications in TE ranging from imaging scaffolds (with or without cells), and preclinical and clinical applications.

Method	Spatial Resolution	Imaging Depth	Information Type	Cost	External Material	Applications
CT	5 µm	No limit	3D	Medium	Yes	Scaffold + cells + Pre/Clinical
MRI	5–200 µm	No limit	3D	High	No	Scaffold + cells + Pre/Clinical
MPI	1 µm	No limit	2D/3D	Medium	Yes	Scaffold + cells + Pre/Clinical
Ultrasound	20–100 µm	10 mm	3D	Medium	No	Scaffold + cells + Pre/Clinical
Fluorescence	0.2–1 µm	0.3–1 mm	2D	Low	Yes	Scaffold + cells
Bioluminescence	2–3 mm	10 mm	2D	Low	Yes	Scaffold + cells
Optical coherence tomography (OCT)	1–15 µm	1–3 mm	2D	Low	No	Scaffold + cells + Pre/Clinical
Photoacoustic	50–150 µm	20 mm	2D/3D	Medium	No	Scaffold + cells + Pre/Clinical
